# Development of a Smartphone App to Predict and Improve the Rates of Suicidal Ideation Among Transgender Persons (TransLife): Qualitative Study

**DOI:** 10.2196/24023

**Published:** 2021-03-24

**Authors:** Alex Dubov, Liana Fraenkel, Zil Goldstein, Hansel Arroyo, Derek McKellar, Steve Shoptaw

**Affiliations:** 1 School of Behavioral Health Loma Linda University Loma Linda, CA United States; 2 School of Medicine Yale University New Haven, CT United States; 3 Department of Medicine Callen-Lorde Community Health Center New York, NY United States; 4 Icahn School of Medicine Mount Sinai Hospital New York, NY United States; 5 Accenture LLC Los Angeles, CA United States; 6 Department of Family Medicine University of California, Los Angeles Los Angeles, CA United States

**Keywords:** mobile health, mHealth, mobile app, pilot study, qualitative research, user-centered design, acceptability study, health services for transgender persons, suicide prevention, mental health, mobile phone

## Abstract

**Background:**

Transgender people are at a high risk of suicidal ideation, suicide attempts, and deaths. Among transgender individuals, 77% and 41% engage in suicidal ideation and suicide attempt in their lifetime, respectively, which exceeds the general population rates (9.2% and 2.7%, respectively). Traditionally, suicide risk factors have been studied over a long period between measurements, making it difficult to understand the short-term variability in suicide risk. Mobile phone apps offer an opportunity to understand the immediate precursors of suicidality through the assessment of behaviors and moods in real time. This is the first study to use a mobile phone app (TransLife) to understand the short-term risk factors for suicide among transgender individuals.

**Objective:**

This study aims to beta test the usability of an evidence-informed mobile health (mHealth) suicide prevention phone app, TransLife. The primary aims are to obtain preliminary data on user engagement and satisfaction with the app, and to assess the feasibility of completing ecological momentary assessments (mood logs) within the app.

**Methods:**

We used qualitative methods and an exploratory research approach that combined naturalistic app use, focus groups, and semistructured phone interviews. The focus group was informed about the development of the prototype. We conducted a 3-week evaluation to determine engagement and obtain detailed user feedback about the app. After participation in the pilot, phone-based, semistructured, and audio-recorded exit interviews were conducted with the research participants.

**Results:**

In total, 16 transgender individuals participated in this study. On average, users logged in 4 (SD 2.7) times a week and spent approximately 5 (SD 3.5) minutes on the app per log-in. A total of 6 major themes emerged in this study. These themes focused on the app’s functionality, satisfaction with using the app, perceived ease of use, perceived safety of providing personal data within the app, trusting the app enough to share personal feelings, and features that make this app engaging. These themes suggest that TransLife is an engaging, useful, and acceptable mHealth intervention. Participants reported that the app was easy to use and understand, supported mental self-care, promoted self-awareness, and helped them identify triggers of negative moods.

**Conclusions:**

The results of this pilot study indicate that TransLife is an engaging, acceptable, and potentially effective mHealth intervention. Transgender participants reported many advantages of using TransLife, such as being able to track their mood, connecting to the community, and accessing local resources. This study provides initial support for the acceptability and usability of TransLife as an mHealth intervention designed for the transgender community.

## Introduction

### Background

Transgender people, who represent up to 0.5% of the adult population, have an extremely high prevalence of suicidal ideation (SI; 77%) [1] and suicide attempts (SAs; 41%) [2]. The rate of lifetime SI and SAs exceeds that of the overall US population (9.2% and 2.7%, respectively) and the lesbian, gay, and bisexual population (10%-20%) [3]. The majority of previous research on SI and SAs among transgender people has focused on the same sociodemographic (sex assigned at birth, employment, age, and ethnicity) and psychiatric risk factors (eg, depression) for suicide. However, these factors are not specific to SI and SAs, do not indicate when an individual is at increased risk, and are removed in time from an actual SA. Furthermore, existing studies of SI and SAs among the transgender population have a number of methodological limitations in how studies define transgender people (eg, exclusion of nonbinary) or measure SI and SAs (eg, a mixture of validated and researcher-designed measures) and in their mostly cross-sectional nature. Current models of suicidal behavior among transgender people have a limited ability to predict suicide risk. Improved methods to identify transgender persons at a high risk of SI are urgently needed.

Recent research has demonstrated that SI fluctuates over time and varies considerably within each person [4]. From a prevention perspective, it is crucial to understand the factors that are the strongest predictors of SI in the weeks, days, and hours before an SA. Long-term risk factors of SAs, described in the literature, are poor predictors of short-term risk [5]. This is one of the most significant limitations of the existing studies, namely, their tendency to measure SI risk at a single point in time, covering long retrospective periods. Currently, no studies have proposed an understanding of short-term modifiable risk factors among the transgender population. To address this limitation, it is necessary to capture new, dynamic, and real-time data on immediate risk factors for SI. Mobile technology allows researchers to augment standard surveys and clinical examinations with dynamic, real-time data. Ecological momentary assessment (EMA) embedded in phone apps offers the opportunity to understand the immediate precursors of SI in daily life by assessing behaviors, experiences, and mood states in real time.

### Objectives

This study aims to beta test the usability of an evidence-informed mobile health (mHealth) SI prevention phone app, TransLife. The TransLife app provides transgender people with resources to promote resilience and cope with minority stressors from the Meyer Minority Stress framework [6]. The framework is based on the premise that the experiences of stigmatization (eg, prejudice, concealment, self-stigma, expectation of rejection) take the form of a specific minority stressor, which in turn affects the state of health of a transgender person [7]. The TransLife app also enables longitudinal data collection that can be used to create a predictive model of SI risk among transgender people. Before we finalized the development of the TransLife, we conducted a focus group with several transgender community leaders to ensure usability. This paper describes the development of the TransLife app and its features and the results of beta testing research. Beta testing aims to test software with end users in a real-world setting to identify and rectify any potential issues before being released. To ensure that a mobile app is useful, experts recommend that it should be evaluated in terms of its utility (whether an app provides the features the users need) and usability (how easy and pleasant the features are) with users. In this study, we aim to obtain an in-depth understanding of users’ acceptance of the TransLife app to monitor moods and feelings, track health activities, and connect with the broader community.

## Methods

### App Development

A multidisciplinary team of software engineers, transgender health professionals, mHealth specialists, and psychiatry specialists created an initial version of the TransLife app. This team carried out an iterative [8], user-centered [9] design process to develop the TransLife app prototype with periodic input on concepts, features, and measures from the Transgender Health Program team of St. John’s Well Child and Family Center in Los Angeles. All St. John’s team members identify as transgender and represent target end users, in addition to bringing a wealth of experience in providing health and social services to more than 1400 transgender patients. Once a prototype app (version 1.0) was completed, it was further tested in a focus group discussion with staff and clients of St. John’s Transgender Health Program to identify preferences and requirements to consider including in the TransLife. Their feedback was incorporated into the design of version 2.0 beta tested for usability and acceptability in this study.

### The App

The TransLife app has several components: *Dashboard* is the landing page of the app. It provides users with updates and information regarding their progress, surveys that need to be completed, upcoming events, and reminders. It offers important notifications and announcements to help users use apps more effectively. It also dynamically suggests user resources and activities based on their mood patterns collected through the app’s usage. For instance, if the mood has been persistently negative for the past 3 or more days, the app will recommend taking a survey (brief measures of depression and SI) typically administered within 2-week intervals. Users will also receive practical tips on coping with negative moods and positive reinforcement when their mood improves. *Mood logger* allows users to log their mood, providing data regarding the events in a user’s life and the corresponding effect on their mood. Users can access their long-term mood tracking data within the app, whereas researches collect the same well-structured data on the backend. Users can record their general mood, several related emotions, and potential reasons for this emotional state (together with their notes). This feature design is intuitive, colorful, and easy to use. Users are invited to log their sleep quality and energy levels. Users and researchers can create and publish *events*; they can search through the list of useful resources (health providers, services, etc) vetted by other app users in the user’s vicinity on the *local* page (similar to the Yelp feature). The *Insights* tab displays a log history of the user’s mood. Logs can be seen by week, month, and year to track progress. This feature provides a graphical representation of moods to quickly assess progress. Minimal demographic data were collected during the sign-up process to streamline the first experience with the app. The *Surveys* feature allows researchers to gather more relevant demographic data in addition to periodic monitoring of users’ depressive moods and SI (at minimum every 2 weeks, when a reoccurring reminder is pushed to the user). Finally, there is a list of *resources* under a dedicated tab divided into health care and mental health resources, housing and legal resources, and resources for victims of violence. This feature has a comprehensive search and filter option to help the user get to the topic they need.

### Safety Features

We ensured that the app and related data could be completely wiped from a device when the participant leaves the study, and we also provided assurances to the participant. All secondary agreements (eg, run-time libraries, standard services provided by the carrier) that collect and send data to third parties were identified and evaluated for risk. Files and data stored by the app were automatically encrypted whenever the device was locked. The latest version of secure socket layer (SSL; version 3.0) / transport layer security (TLS; version 1.3) encryption protocol was used for all communications between the app and other systems, including user authentication and the transfer of sensitive information. Data were stored and backed up using encrypted, password-protected storage services provided by the university.

Safety planning is a clinical intervention component often included in the treatment of suicidal patients and specifically aimed at the transition from having the thoughts or intention to engage in an SA and acting upon the thoughts or intention [10]. The use of safety planning is recommended for patients at risk of SAs in suicide prevention guidelines [11], and safety planning is embedded in cognitive behavioral therapy (CBT) for suicide prevention [12]. Our safety plan conforms to the best practices [13] and includes the following steps: (1) identifying early warning signs of an impending suicidal crisis (eg, negative feelings and problematic behaviors), (2) employing internal coping strategies, (3) employing distraction activities and socialization to distract from SI, (4) making use of social support contacts who may offer help, (5) collating the contact details of mental health professionals and other crisis resources, and (6) making the environment safe. The Safety Plan feature is a part of onboarding new users, together with informed consent. The Safety Plan feature is stored in the app.

### Study Design

This pilot study used qualitative methods and an exploratory research approach that combined naturalistic app use, focus groups, and semistructured phone interviews. The primary aims were to obtain preliminary data on user engagement and satisfaction with the TransLife app and to assess the feasibility of completing EMAs (mood logs) within the app. To achieve these aims, we conducted a 3-week evaluation to determine engagement and obtain detailed user feedback about the app. After participation in the pilot, phone-based, semistructured, and audio-recorded exit interviews were conducted with 10 transgender participants. All interviews were transcribed and independently coded by 2 study team members. Content analysis was conducted to identify data categories and overarching themes.

### Recruitment

As not all transgender individuals may publicly identify as such, it is difficult to recruit a sample using traditional techniques. One approach that has shown promise in sampling hidden populations, such as transgender people, is respondent-driven sampling (RDS) [14]. RDS relies on chain recruitment but limits the number of individuals who can be recruited by each participant. Eligible participants were recruited through transgender key informants (1 transgender woman and 1 transgender man). These informants were invited to recruit 2 additional participants. The participants in the first wave were asked to invite up to 2 peers to participate. Participants were recruited between May and June 2019. Eligibility criteria included self-identifying transgender people aged ≥18 years, living in the Greater Los Angeles area, and owning a smartphone. Interested participants were asked to complete an electronic consent form and download the TransLife app from the TestFlight (a tool that allows developers to test an app before it goes on the web on the app store). Once the app was installed onto their phone, participants were asked to click on different app features to become familiar with their functions. Participants were encouraged to use the app during the 3-week trial period; however, there was no required amount of time to use the app during the trial. Participants were compensated at the end of the trial period with a US $20 virtual Amazon gift card. The Loma Linda University Institutional Review Board approved this study.

### Procedures

The System Usability Scale (SUS) [15] and the Standardized User Experience Percentile Rank Questionnaire (SUPR-Q) [16] are 2 validated and widely used measures of user experience. SUS is an industry-standard scale used to evaluate a variety of products and services, including websites, mobile phones, and computer software. SUPR-Q is a well-validated 8-item instrument that measures the quality of a website’s user experience. Questions from both instruments were used to inform the development of the interview guide for this study. The final interview guide consisted of 12 items (Multimedia Appendix 1). Semistructured, phone-based, and audio-recorded interviews were conducted with 16 participants. Nielsen et al [17] showed that conducting usability testing with only 5 participants will reveal 85% of usability problems. All interviews were conducted by one investigator (AD) who had experience with qualitative research. The interviews were transcribed and supplemented with the previous focus group discussion transcripts. Data saturation was perceived through recurring ideas, such as convenience of use, motivation to use the app, engagement with the app, and ease of use.

### Data Analysis

We used a 6-step thematic analysis approach [18] to capture the user experience themes. For step 1, we familiarized ourselves with the transcripts. For step 2, we confirmed the selection of codes and themes and made necessary amendments to reach a consensus. Initial coding was performed in Atlas.ti based on the deductive coding framework, with varied responses interpreted inductively into new codes as needed. Codes were matched between authors to ensure consistency and confirm the definition of the full set of themes. In step 3, we clustered nodes into a common theme based on coherent patterns. We used some of the quotes in the Results section to demonstrate the legitimacy of the defined themes. For step 4, we reduced the themes into the most prevalent implicit and explicit ideas while deleting redundant themes. In step 5, we described the names and parameters of the identified themes. For step 6, we reported the analysis performed. In total, 6 major themes comprising several categories and codes emerged. These themes focused on the app’s functionality, satisfaction with using the app, perceived ease of use, perceived safety of providing personal data within the app, trusting the app enough to share personal feelings, and features that make this app engaging. We applied methodological data triangulation by comparing data sources, namely, qualitative individual interviews and focus groups. Triangulation enhanced data richness or completeness by enabling researchers to explore a broad range of perspectives and compare and contrast perspectives on the usability and feasibility of the TransLife app [19]. The multidisciplinary team involved in the analysis helped to minimize the risk of bias because of a variety of expertise and interests by challenging each other’s assumptions and interpretations.

## Results

### Demographic

A total of 16 transgender individuals participated in this study. Participant characteristics are shown in Table 1; participants’ mean age was 33.4 (SD 9.5) years. Half (n=8, 50%) of the participants were identified as male-to-female transwomen, 37% (n=6) as female-to-male transmale, and 12% (n=2) of participants were identified as third gender (other). Participants were White (n=7, 44%), Hispanic or Latina (n=5, 31%), African American (n=2, 12%), and Asian (n=2, 13%). Most participants were highly educated (n=11, 69% of college graduates), and 63% (n=10) were employed full time.

**Table 1:** Demographics (N=16).

Demographic		Value	
Age (years), mean (SD)		33.4 (9.5)	
**Gender identity, n (%)**			
	Male to female	8 (50)	
	Female to male	6 (37)	
	Other	2 (12)	
**Race or ethnicity, n (%)**			
	White	7 (44)	
	Hispanic or Latina	5 (31)	
	African American or Black	2 (12)	
	Asian or Pacific Islander	2 (12)	
**Education, n (%)**			
	High school		1 (6)
	Some college		4 (25)
	College graduate		7 (44)
	Postgraduate studies		4 (25)
**Employment status, n (%)**			
	Employed (full time or part time)		10 (62)
	Student		3 (19)
	Unemployed		3 (19)

### System Usage Data

On average, users logged in 4 times a week and spent approximately 5 minutes on the app per log-in. The mood logger and question of the day were the most used features of the app. It took participants, on average, one and a half minutes to log their moods.

#### Engagement

Although the adoption of an app is important, users’ continued interaction with an app (engagement) is key to user retention. According to a recent survey, 25% of installed mobile apps are never used, and 26% of installed mobile apps are abandoned after the first use [20]. The literature defines engagement as a psychological process that leads to the formation of loyalty [21] or the intensity of an individual’s participation in and connection with app features or activities [22]. Most participants reported that they found TransLife to be helpful and engaging through its features and offerings. Participants noted some improvements in their moods and growing self-awareness as the result of using TransLife, as illustrated by this quote:

This app helped me to get in touch with my moods. At times I would stop and think – how do I feel now? Having to identify my feelings and emotions has helped me to become a bit more self-reflective.

Similarly, another participant commented that the persistent use of the app helped him gain a better understanding of his mental health:

After about ten days of use, it has become clear that my mood is usually better next day after going to the gym the previous afternoon...there also seems to be a correlation between my low moods and unhealthy food choices...I definitely feel better when I am more active. Great insights from using this app.

Some participants mentioned that monitoring their mood has helped them regain control over their mood states and offered some measure of self-efficacy:

Mood logging made me more aware of my feelings, the way my mood changes over a period of time, and how to deal with mood changes. Even when I feel down, I can remind myself that these feelings are normal and will likely pass.

Participants noted several features of the app as driving engagement and promoting self-insight. The participant’s comment on using the insights feature that provides a graphic representation of mood history is as follows:

Insights was one of the most helpful features. It was interesting to see how your moods change over time and progressions of either positive or negative feelings.

During the design process, we added several features with the sole purpose of promoting engagement, such as transgender-specific news or questions of the day polls. The question of the day allows users to respond to a particular question and then compare their answers with peer responses. These app features were successful in driving engagement, as illustrated by the following quote:

Question of the day was something that kept me engaged with the app. I really liked seeing the answers from other folks and I can only imagine how interesting it would be to check the answers once the app will add more users.

#### Functionality

The functionality feature of an app refers to the user’s perception of different functions within an app and how these functions are effective in reaching the user’s goals [23]. Functionality usually refers to more tangible physical features, such as layout, navigation, and esthetics. Participants described the interface as intuitive and straightforward:

I like the general interface and how well the app is organized. I think it is very well designed. I used mood logs every day and found it very helpful.

Although commenting on the availability of similar mood tracking apps, several participants mentioned their intention to switch to TransLife when it becomes available within the app store. Several unique functions of the app, coupled with the overall transgender specificity of the design and content, were the main reason participants were considering switching to TransLife. Participants commented on the value of a culturally tailored mental health app for the transgender community:

To be honest, I’ve been using another mood tracking for the past several months. I would consider switching to this app when it is available in the app store because of several reasons. This app is trans specific. For example, you can add “gender dysmorphia” as one of the reasons for your bad mood. Also, this app has more to offer than just a simple mood tracker – a version of yelp, resources, even trans news.

Participants appreciated the ability to customize the app’s appearance and its functionality to fit the participants’ personal needs. Participants liked to customize alerts and reminders to fit individual daily routines, for example:

I like the idea of being able to customize alerts and reminders from the get-go. It is so useful that the app can remind you about your meds. We used to do it with my friends, reminding each other about our meds. The mood logging reminders are also customizable, and I like that.

#### Privacy and Security

Mobile apps offer tremendous potential to enhance mental health, but they often lack data security and privacy mechanisms needed to increase user’s and clinician’s confidence in these apps. Low confidence in an app’s ability to provide a private and secure environment is a significant barrier to its adoption. Concerns about the security and privacy of the information provided on the app were evident among some participants:

I had some difficulty answering question of the day as I don’t like putting too much information about myself out there. Maybe I am old-fashioned that way.

However, this concern about privacy and security was not shared equally among all participants. Several app users discussed their lack of concern about data security and their comfort when using a mood diary, leaving notes, and answering survey questions:

This may sound weird, but I rarely think about data security, to be honest...It is rarely my concern, but I guess it should be.

Several participants described the potential for collaboration between TransLife users and their therapists and their comfort with sharing mood logs and notes with health providers. Participants linked the possibility of sharing personal information with the benefits of receiving help and enhanced coaching from their therapists:

I am currently working with a therapist, and I was really excited to see the insight feature. There were times when I would come to my therapist and tell him that my week was awful. I was rarely able to be more precise than that, and we would spend the whole session trying to dig out my triggers. Now I can bring my diary and just show it to him.

#### Ease of Use

Users of TransLife described the app as being easy to use, accessible, simple, and easy to navigate. Participants appreciated the ease of use and convenience of the TransLife app:

I don’t see how anyone would have issues with navigating this app. It is well-structured and eases you into things.

The simplicity of the app and its intuitive design was highlighted as aspects likely to promote the continued use of the app. Simplicity, as indicated by a user-friendly and intuitive design, was considered a positive attribute of the app:

I believe the app was, in general, very intuitive and easy to use. There was nothing that would confuse me or made me pause and think how to navigate it. This is the main reason I want to use it in the future.

The onboarding process is a participant’s first impression of the app. When it is designed correctly, it increases the likelihood of app adoption. Conversely, a complicated onboarding process may result in users not understanding how to use the app, thus having a negative experience and possibly abandoning it after the first use. Participants mentioned that their onboarding process was easy, simple, and straightforward:

I don’t remember much from the registration process and setting up my account. That is probably to say that the whole process was easy and painless.

#### Trust

Trust is an essential component of mHealth adoption. In general terms, trust can be defined as the willingness of a person in a position of vulnerability to take a risk despite uncertainty, placing confidence in the intentions and competence of another. Users of an mHealth app need to trust the app and its creators to provide meaningful content. To assess participants’ comfort with the app and to trust the app with deep thoughts or emotions, we looked through optional free-text notes left together with mood logging. There were many instances when participants shared events from their lives, along with their emotional reactions to these events. For example, one participant described his anxiety about seeing a physician:

I’ll probably have to go to the doctor tomorrow...I hate going to the doctor as it disrupts my whole day, and I find the experience to be overall stressful, so I’m feeling annoyed at myself for being sick. I hate being sick, especially when it just doesn’t go away on its own, and I feel like I have to ask for help and get doctors involved.

Another participant anxiously anticipated her trip back home after being gone for a long time. She was expecting a negative reaction from her parents to her appearance, and she was mentally preparing to face criticism:

Worried about my upcoming trip with my family and feeling very overworked trying to afford plane tickets. I’m hoping to avoid criticism, but it’s really inevitable. Maybe things will get better once we have the chance to talk more. It’s been a long time.

Participants shared not only their worries and fears but also their positive emotions, such as this entry from a user who was excited about the upcoming pride weekend:

Today is the start of my pride weekend. I just really want to have such a good time. I want to drink a lot and look real cute and run around feeling free and happy...I want to make new friends and run into old ones. I want everyone to like me and want to talk to me and flirt with me and tell me how good I look and how much fun they are having being around me.

Even more mundane notes, not linked to a significant event such as pride weekends or a trip back home, were still insightful and demonstrated participants’ trust and comfort with using the app for self-reflection:

A rare night of uninterrupted sleep. Yet I feel pretty tired and emotionally drained this AM. Hoping the sunshine, mild temps, and productive day will boost my mood and energy.

#### Satisfaction

Several studies [24,25] link app continuance intention with user satisfaction, defined as “an overall affective response to the gap between prior expectation and perceived performance after consumption.” One of the accepted ways to measure user satisfaction is to assess users’ willingness to recommend the app to others. Participants were willing to mention this app with their friends:

I feel like I’ve already mentioned this app to friends. Even considering the issue of finding a care provider. This is something that a lot of people are struggling with – finding a friendly and knowledgeable provider. If this app could help them with this while also tracking their moods and connecting them to a wider community – this would be amazing!

There is a connection between user satisfaction and the perceived value of the app. Most participants viewed the app as useful and connected their satisfaction with TransLife to the benefits of using it:

I rated this app so high because I really like the idea of it and I think it is very helpful for a lot of people. It is perfect for finding trends in your moods and for becoming more self-aware.

Many participants noted the potential for this app to improve and the fact that the new release of TransLife used by a large number of people will make the app especially beneficial. Several features of the app, such as a version of Yelp called local or *question of the day* feature, rely on user-generated content. Participants appreciated the potential for these features to become even more useful and engaging, making the TransLife app even more satisfying to use:

I would give this app four out of five because it has room to grow – more resources can be added, more local content. This app has the potential to become a very useful tool for a lot of people I know.

## Discussion

### Principal Findings

This study was the first to explore attitudes toward mental health app use among a diverse sample of transgender adults. The purpose was to discover what components or features of a mobile mental health app may resonate with transgender persons and what concerns and suggestions they may have about a mental health app. This addressed an important gap in the literature, as we sought input regarding mental health apps from this priority population with high rates of SI and SAs. The overall findings revealed that transgender people are quite enthusiastic about the role of mobile apps for mental health support and community building. More specifically, we sought to explore the acceptability, use, and feasibility of a new smartphone app intervention, TransLife. Themes that emerged from our analysis suggested that TransLife is an engaging, useful, and acceptable mHealth intervention. Participants reported that the app was easy to use and understand, supported mental self-care, promoted self-awareness, and helped them identify triggers of negative moods.

### Acceptability

Mood tracking was well accepted and tolerated by participants. We found that participants wanted to learn about their mood patterns and improve their ability to manage emotional distress more efficiently. Participants were willing to pursue these goals through the regular mood tracking feature, through mood data visualization using the insights feature, through registering contextual information in mood notes, and being willing to share this information with health care providers. The acceptability of this app is enhanced by designing app experience with the transgender population in mind (eg, addressing unique mental health needs) and offering flexible and personalized mood tracking options (eg, integrating contextual information, ability to customize reminders). Acceptability can be further enhanced by increasing app design to be private, discrete, and confidential to use. Participants recognized the potential for mood tracking to facilitate earlier identification of mood changes and enhance clinical care. They also valued the opportunity to view and make sense of their mood data to gain a more individualized and accurate picture of their mental health. This, in turn, presents the opportunity for more dynamic and flexible service provision and monitoring of treatment outcomes.

### Usability

Participants thought TransLife was accessible, easy to navigate, and not burdensome. Ease of use is essential in facilitating engagement, and TransLife was noted for being user-friendly and intuitively designed. In addition to regular engagement with the mood logger, many participants chose to write notes in the associated diary without specific prompts, indicating a level of trust in the app. Participants used mood tracking and diary to reflect on their emotions and develop awareness about what may influence positive or negative moods. The question of the day feature sustained engagement and created a community feeling within the app, whereas events and local features connected participants to their local community and helped them identify local resources. Many participants were not engaged in mood tracking daily (4 days per week on average, SD 2.7). When asked to suggest features that would support sustained engagement, participants provided feedback that would be incorporated into the next release of the TransLife. Participants recommended including a transgender-specific news stream, gamification feature, and tailored activity suggestions based on mood (eg, coping strategies).

Although existing studies have highlighted the disparities in mental health outcomes between transgender people and cisgender people, most of this research has examined transgender people as a single group. Given the limitations of this study, we cannot conclude the mental health status and treatment-seeking behavior of various subgroups within the transgender community. However, we noticed several trends among our study participants, suggesting a lack of uniformity in dealing with psychological stressors and using self-help tools. Nonbinary participants reported a previous history of SAs when taking the baseline survey; they made fewer mood entries but often used *anxious* *frustrated* tags, and their engagement with the app was minimal. Transgender men more frequently made use of the free-text entries associated with mood logs, engaged with the app resources more often, and actively suggested app improvements and additional resources. Notes and mood logs of transgender men indicate a higher level of gender dysmorphia. Transgender women frequently reported a history of depression in their baseline surveys and mood logs. During their interviews, transgender women talked about a lack of access to transfriendly mental health services, and they welcomed an app designed with their community in the view that it would equip them with self-care tools for mental health and connect them to transfriendly services.

### Limitations

This exploratory pilot study has several limitations. First, the sample size and study design (eg, naturalistic app use and qualitative interviews; n=16), albeit consistent with recommendations for early pilot intervention work [26], does not allow for strong causal conclusions. Second, although this study was useful for gaining insight into the transgender people's experience and perceptions of the app over 2 weeks, it would be valuable to examine whether these perceptions change over a more extended period of naturalistic use. Third, our participants were highly educated, had stable housing and employment, and lived in urban areas. Therefore, these results may not be generalizable to all transgender populations.

### Broader Applicability of Findings

#### Need for Transgender-Specific Apps That Provide the Community With Resilience-Building Resources

Several mood-logging apps and CBT mHealth interventions have been developed for the general population. These interventions are not tailored for transgender people; they do not address stressors unique to transgender people (eg, ability to log *gender dysmorphia*), and they were not designed to collect longitudinal data. Furthermore, currently, there are no apps designed specifically for transgender people to help them connect with the community of peers, access a variety of community prevented resources, and access transgender-specific health care resources. TransLife is the only app that is tailored to the transgender population and provides them with resources, connects them to their communities, and monitors their psychological well-being.

#### Need for a More Complex Understanding of Transgender SI Risk

The need to identify modifiable immediate predictors of SI is urgent and widely acknowledged but rarely accomplished because of small sample sizes and methodological limitations. Most studies on transgender SI risk are cross-sectional, collecting data at a single point in time. Although they are able to demonstrate that certain SI risk factors tend to co-occur with reports of past SAs, they are not able to provide evidence that certain risk factors are causally related to earlier behaviors. As SI is associated with SAs, there is a need for prospective data that will allow us to differentiate between risk factors that cause initial SI and risk factors that prolong its duration. The ability to monitor high-risk transgender participants using EMA measurements embedded in TransLife, and the potential to detect certain behavioral or environmental influences and use them as markers of elevated risk may prove to be very meaningful in understanding the short-term risk of SI.

#### Identification of Modifiable Risk Factors Using EMA

Currently, there have been no studies using EMA to understand short-term modifiable SI and SA risk factors among the transgender population. EMA offers the novel possibility of providing information about the short-term correlates and predictors of SI and SAs, thereby improving our understanding of the suicidal process, detecting persons at risk for SI, and fostering the development of effective strategies for suicide prevention. However, existing studies using EMA mostly focused on negative mood alone while discounting many social, environmental, and behavioral variables. TransLife uses a variety of EMA measures to assess not only negative mood but also behavior, environment, social context, and daily life events.

### Conclusions

This is the first study to investigate the acceptability of a mental health app among transgender individuals. The results of this pilot study indicate that TransLife is an engaging, acceptable, and potentially effective mental health intervention. Transgender participants reported many advantages of using TransLife, such as being able to track their mood, connecting to the community, and accessing local resources. This study provides initial support for the acceptability and usability of TransLife as an mHealth intervention designed for the transgender community. The next steps involve further app refinement and more substantial feasibility testing. A large-scale randomized controlled trial is warranted to evaluate the efficacy of TransLife on mental health outcomes among the transgender population.

## Appendix

Multimedia Appendix 1 Interview guide.

## Abbreviations

CBT: cognitive behavioral therapy
EMA: ecological momentary assessment
mHealth: mobile health
RDS: respondent-driven sampling
SA: suicide attempt
SI: suicidal ideation
SUPR-Q: Standardized User Experience Percentile Rank Questionnaire
SUS: System Usability Scale
