# Supplementary material for: Development of a Smartphone App to Predict and Improve the Rates of Suicidal Ideation Among Transgender Persons (TransLife): Qualitative Study
Source: J Med Internet Res. 2021 Mar 24;23(3):e24023. doi: 10.2196/24023 (PMC8074983; doi:10.2196/24023)
Supplement: Multimedia Appendix 1 [file jmir_v23i3e24023_app1.docx]

**Interview Guide**

1. Did you experience any issues in downloading and installing the app? Describe your experience with creating your user profile and completing the baseline survey.
2. How easy or difficult was it to use the app to complete mood tracking?
3. I’d like to know how long it took you to do the daily mood tracking. Was it too long, too short, too cumbersome?
4. How often would you prefer to track your moods and why? Daily, few times a week, weekly?
5. Did this app help you get in touch with your moods and feelings?
6. I want to discuss with you several features of this app...*Dashboard*—How easy was to navigate the dashboard? *Question of the day*—Did you enjoy learning from other users? *Quote of the day*—Did you find quote of the day inspirational? *Insights*—Did you review the insights? *Resources*—Is it helpful to have resources readily available on the app?
7. Was the layout and design of the app intuitive enough?
8. How satisfied were you with the app overall?
9. Were you concerned about privacy of your information while using the app?
10. What features of the app did you find most useful? Less useful? If there is one thing you could change about this app, what would it be?
11. How likely would you recommend the next version of this app to a friend (given the app will be improved based on your feedback)?
12. How likely would you participate in the pilot testing of the v2 of the app in the feature?
